# Supplementary material for: Transcriptomic Insights into Mechanisms of Early Seed Maturation in the Garden Pea (Pisum sativum L.)
Source: Cells. 2020 Mar 23;9(3):779. doi: 10.3390/cells9030779 (PMC7140803; doi:10.3390/cells9030779)
Supplement: Supplementary file 1 [file cells-09-00779-s001.zip › Supplementary materials/FileS2.zip!/FileS1/Summary Sheets - PATs/PAT report.pdf]

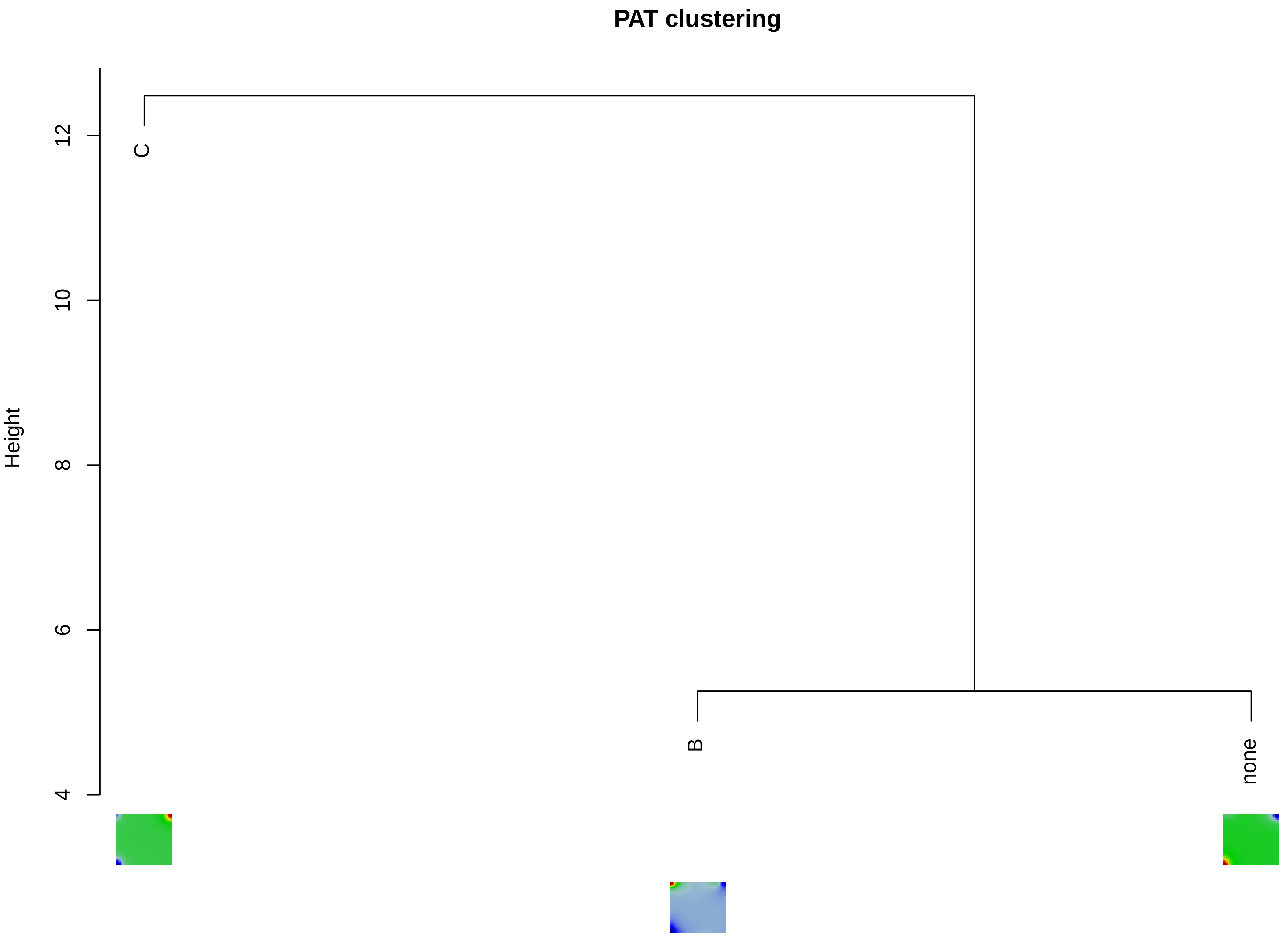
PAT clustering
Height
12
10
8
6
4
C
B
none

none

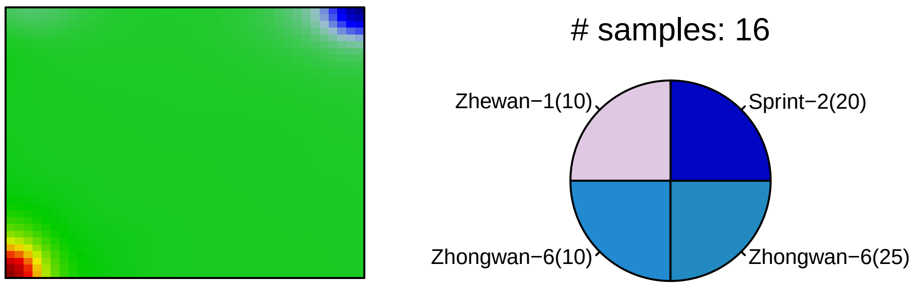

samples: 16
Zhewan−1(10)
Sprint−2(20)
Zhongwan−6(10)
Zhongwan−6(25)

B

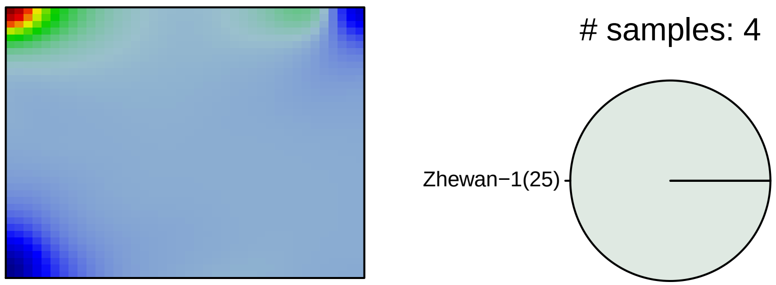

samples: 4
Zhewan−1(25)

C

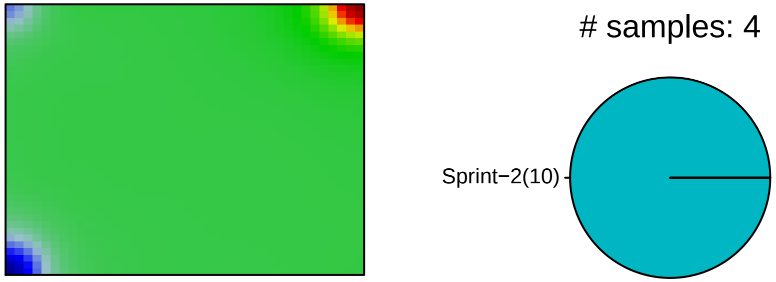

samples: 4
Sprint−2(10)

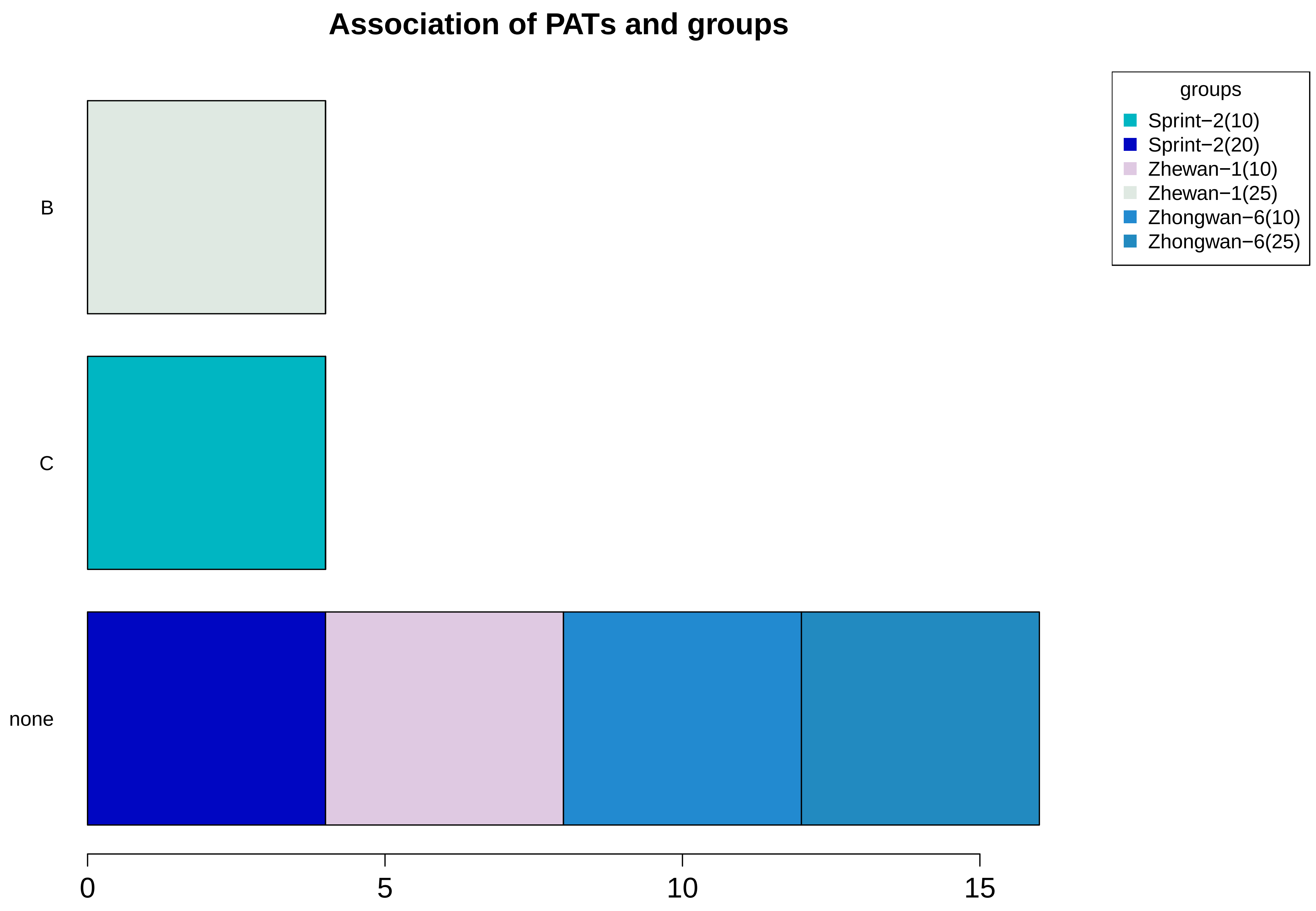
Association of PATs and groups
B
C
none
0
5
10
15
groups
Sprint−2(10)
Sprint−2(20)
Zhewan−1(10)
Zhewan−1(25)
Zhongwan−6(10)
Zhongwan−6(25)

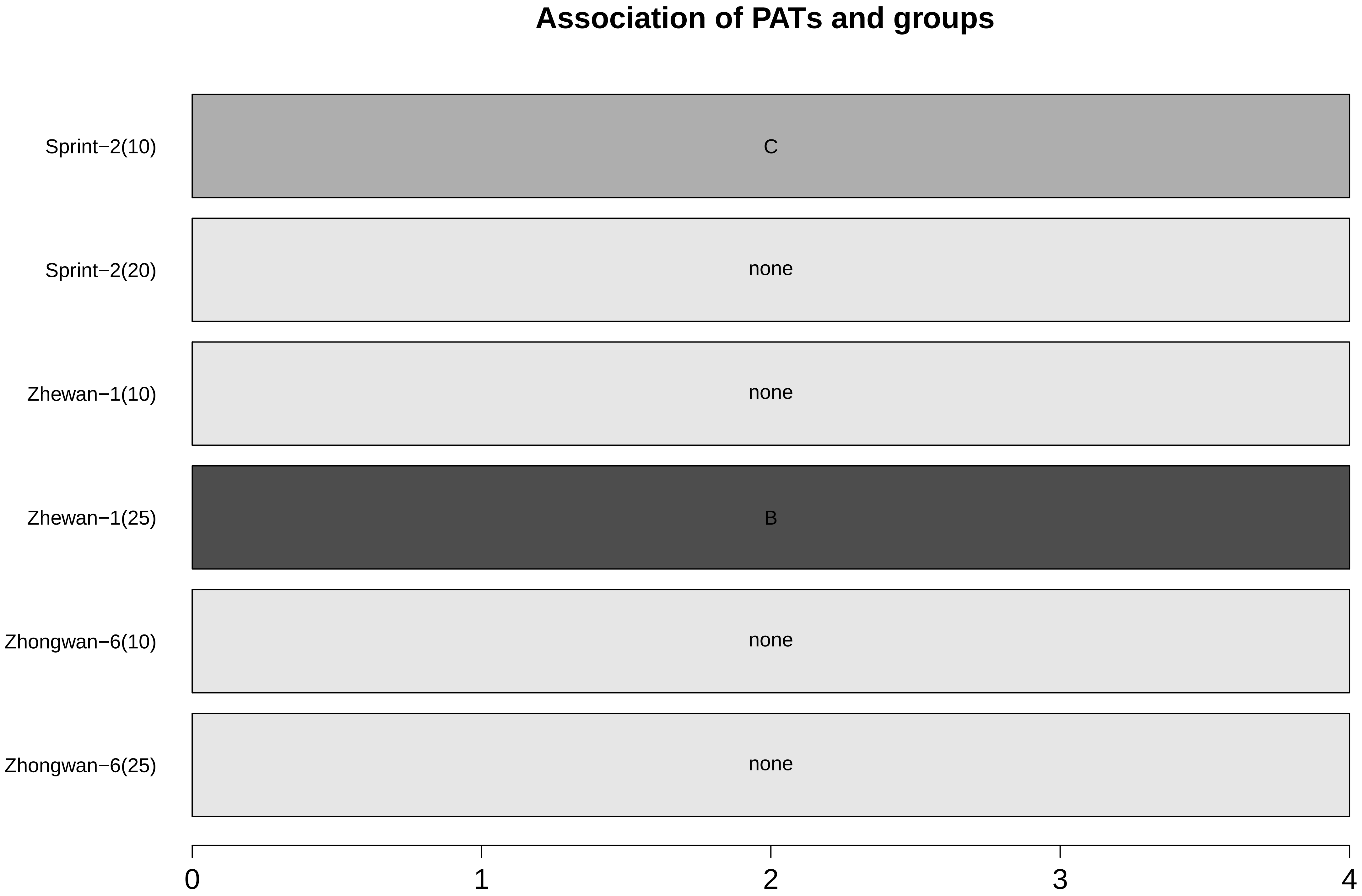

Association of PATs and groups
Sprint−2(10)
C
Sprint−2(20)
none
Zhewan−1(10)
none
Zhewan−1(25)
B
Zhongwan−6(10)
none
Zhongwan−6(25)
none
0
1
2
3
4
